# Arising Concerns of Atypical Manifestations in Patients with Hand, Foot, and Mouth Disease

**DOI:** 10.3390/vaccines11020405

**Published:** 2023-02-10

**Authors:** Yu Chen, Bowen Dai, Shujie Han, Guangcai Duan, Haiyan Yang, Shuaiyin Chen, Wangquan Ji, Yuefei Jin

**Affiliations:** 1 Department of Epidemiology, College of Public Health, Zhengzhou University, Zhengzhou 450001, China; 2 Academy of Medical Sciences, Zhengzhou University, Zhengzhou 450001, China

**Keywords:** HFMD, atypical manifestations, CVA6, differential diagnosis, genetic evolution

## Abstract

Hand, foot, and mouth disease (HFMD) is a mild exanthematous, febrile disease, but it also remains a threat to global public health. HFMD is characterized by a brief febrile illness in children and with a typical skin rash of the hand and foot, with or without mouth ulcers. However, the morphology and distribution of vesicles, as well as accompanying symptoms, are varied among atypical HFMD. An upsurge in atypical presentations of HFMD caused by *Coxsackievirus* A6 (CVA6), including Gianotti–Crosti-like eruptions, eczema coxsackium, petechial/purpuric eruption, and vesiculobullous exanthema, can be difficult to diagnose clinically as it may mimic other severe skin diseases, such as eczema herpeticum, varicella, disseminated zoster, and erythema multiforme major. The recognition of the distinguishing features of atypical HFMD is vital for an accurate and timely diagnosis, as is initiating appropriate laboratory evaluation and supportive care. Clinicians must identify the wide range of cutaneous and mucosal alterations caused by atypical HFMD. A systemic, high-quality overview of atypical HFMD is needed for advances in better strategies for clinical diagnosis and treatment. Hence, this review is aimed at summarizing the available data on clinical investigations and differential diagnostics to provide a scientific guide for the timely diagnosis of HFMD for preventing serious complications.

## 1. Introduction

Hand, foot, and mouth disease (HFMD) is a highly contagious, globally epidemic disease that primarily affects children under 5 years old [1]. Apart from general symptoms such as fever, the most typical symptom is a rash on specific areas such as the hands, feet or/and mouth [2]. Currently, HFMD has received great attention due to increasing evidence that the clinical, epidemiological and etiological features of the disease are significantly different from those initially thought [3]. Although HFMD is usually a mild disease, neurological complications may occur during large-scale epidemics involving HFMD. A few numbers of patients may develop serious and even life-threatening complications within a short time [4,5]. Moreover, an increasing number of studies have reported that atypical HFMD is becoming a new concern for children’s health [6]. At present, there is no official definition for atypical HFMD, but some studies have defined that it occurs at anatomic sites not listed in the World Health Organization HFMD definition [7]. Many pathogens that can cause atypical HFMD have been reported, such as Coxsackievirus A (CVA) 6, CVA16, CVA4, CVB4, CVB5, and CVB15 [8,9], but particularly CVA6. Currently, CVA6, one of the most prevalent strains in the world; it is also a new variant that has undergone genetic recombination and evolution. Recombination may not only cause variation in the clinical symptoms of HFMD; it may also increase the pathogenicity due to the accumulation of nucleotide mutations [10]. Compared with typical virus strains, the new mutant strains affect a broader population and cause more severe disease with a longer duration [11].

The skin lesions of atypical HFMD in the early stage are very similar to the clinical manifestations of other diseases, such as eczema. However, the early diagnosis of HFMD is mainly based on physical examination, which poses a challenge for clinicians aiming to identify and diagnose HFMD in a timely manner. It can lead to misdiagnosis as well as serious complications, ultimately leading to regional or large-scale epidemics [8]. Therefore, improving the ability of clinicians to identify atypical HFMD is a critical component of clinical practice. The current literature is mostly limited to reports of clinical data but lacks systematic reviews and summaries, especially for differential diagnosis. Therefore, this review focuses on the atypical presentations of HFMD and the differential diagnosis with other similar diseases, providing a scientific basis for the timely diagnosis of HFMD and the prevention of serious complications.

## 2. Literature Search Strategy

Sources consulted for this article included PubMed from the National Library of Medicine, the Centers for Disease Control and Prevention, and the World Health Organization. Search terms included (atypical hand, foot, and mouth disease) OR (atypical HFMD); (atypical presentations/manifestations/characters/clinical features and hand, foot, and mouth disease) OR (atypical presentations/manifestations/characters/clinical features and HFMD). Diseases involved in the differential diagnosis were summarized based on the literature presenting atypical presentation of HFMD. The articles selected for this study were published before December 2022. We also searched for relevant reviews to determine that there were no similar published articles. Then, we performed a manual search of the reference lists from the eligible articles and identified articles to complete our search.

## 3. Inclusion and Exclusion Criteria

The inclusion criteria were: (1) only peer-reviewed, published studies were included, and the subjects were clearly confirmed HFMD cases; (2) the study included atypical clinical features/presentations/manifestations/characteristics of HFMD.

The exclusion criteria of the studies were as follows: (1) abstract-only articles, editorials, duplicated publications, and so on; (2) data and information in the article were incomplete, suspicious, or inconsistent; (3) the full text could not be accessed; and (4) animal experiments.

## 4. Gianotti Crosti-like eruptions

**Figure 1:**
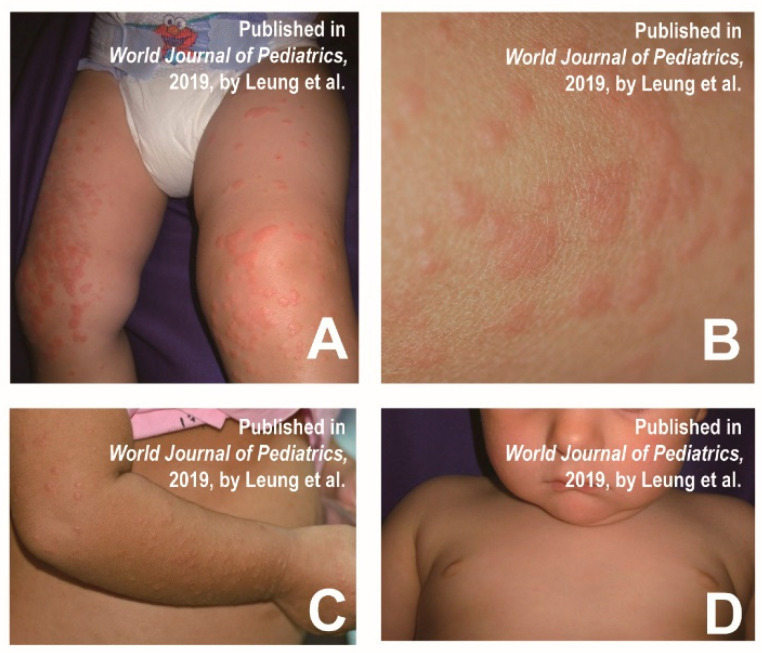
(**A**) Multiple individual, flat-topped, erythematous papules on the external surface of the lower limbs. (**B**) Well-defined and non-squamous papules on the right thigh. (**C**) Multiple monomorphic, flat-topped, erythematous papules on the external surface of the upper limb. (**D**) Monomorphic erythematous papules on the face, with sparing of the trunk. Reprinted/adapted with permission from Ref. [13]. 2019, Leung et al.

Mathes et al. found that a few cases with HFMD had a similar distribution of skin lesions to Gianotti–Crosti syndrome, involving the cheeks, extremities, and buttocks, but the trunk remained unaffected [9]. Typical Gianotti–Crosti syndrome is characterized by monomorphic lichenoid papules and/or papular vesicles, whereas the 2011–2012 North American outbreak was more common in papular sacs with significant erosions. Epstein–Barr virus and hepatitis B virus are the most common causative agents of Crostyanoti’s disease [9], but other viruses (e.g., CVA16, CVB4, and CVB15) are less commonly involved [12]. Diagnostic criteria for Gianotti–Crosti syndrome include the following symptoms: lesions lasting at least 10 days; papules or papular injuries of 1 mm to 10 mm involving three of the following four areas: extensor side of the forearm, extensor side of the leg, cheek or buttocks; and symmetrical distribution of lesions. The clinical presentation is characterized by small, symmetrical isomorphic papules or pimples on the adductor surfaces of the cheeks, ears, arms, and legs, and buttocks (Figure 1A–D) [13]. HFMD patients representing Gianotti–Crosti-like eruptions are more febrile and generally suffer from oral mucosal stomatitis. Papules are more symmetrically distributed on the face, arms and legs, but relatively sparse on the trunk. The rash and blisters are often accompanied with pain and itching.

In addition, Gianotti–Crosti-like eruptions of HFMD may be mistaken for other diseases, such as papulosquamous urticaria and erythema infectiosum [13,14]. Papulosquamous urticaria presents with no fever, groups of or disseminated urticarial pimples, vesicles or wheals, exfoliated pruritus and large blisters developing from small blisters [14]. Erythema infectiosum initially presents as an erythematous rash on the cheeks and can progress to diffuse macular erythema. It is accompanied by paroxysmal pruritus, which is mostly worse at night [14]. In addition, the syndrome is associated with bacterial or viral infections, such as β hemolytic streptococcus, mycoplasma pneumoniae, bartonella aureus, hepatitis A virus, cytomegalovirus, herpesvirus type 6, coxsackievirus, rotavirus, parvovirus B19, and human respiratory syncytial virus.

Once a patient presents similar symptoms to Gianotti–Crosti syndrome, it is required to distinguish the associated causative agents described above. Because the pathogens are different, etiological detection is necessary and effective in order to confirm the diagnosis. The pictures below show the typical signs of Gianotti–Crosti syndrome, which is based on the case reports of 2 children with Gianotti–Crosti syndrome (Figure 1). Table 1 shows the main differential diagnoses of Gianotti–Crosti-like eruptions and some similar diseases.

## 5. Eczema Coxsackium

**Figure 2:**
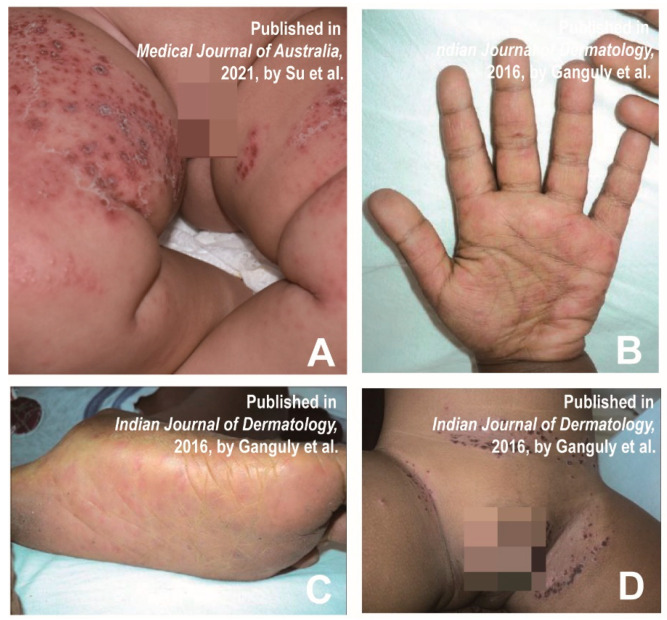
(**A**) Multiple erythematous papules and blisters with crusts on the extremities. Reprinted/adapted with permission from Ref. [23].2021, Su et al. (**B**) Elongated papulovesicles over palm and palmar aspect of fingers. (**C**) Erythematous papulovesicles over soles. (**D**) Crusted papules, vesicles and surrounding erythema and maceration in the groins, abdominal folds, and scrotum. Subfigures (**B**–**D**) were reprinted/adapted with permission from Ref. [21]. 2016, Ganguly et al.

In 1968, a child patient with CVA16 infection presented with a severe widespread papular rash named eczema coxsackium [20]. Eczema coxsackium is clinically characterized by blisters in the area of eczematous dermatitis, which is similar to herpetic eczema caused by the herpes simplex virus (HSV) [21]. In addition, the lesions are more severe at the bends, such as the wrinkled skin of the arms and legs [22]. Most patients have a rash that shows signs of secondary bacterial infection [22]. The skin pattern consists of relatively monomorphic vesicles and/or blisters concentrated in areas that are affected by atopic dermatitis [12]. A girl who was diagnosed with coxsackie eczema presented multiple erythematous papules and blisters around the mouth, trunk and extremities (Figure 2A) [23]. A 3-year-old male patient with multiple elongated blisters on the palms of hands and fingers surrounded by erythema (Figure 2B), as well as on the soles of his feet (Figure 2C) and on his knees, was reported in a previous study. A little erythematous erosion could be seen on the hard palate. Additionally, numerous partially fused crusted papules with a few blisters surrounded by erythema and maceration were observed in the groin, abdominal crease, and scrotum (Figure 2D) [21]. 

As eczema coxsackium resembles herpetic eczema, overlapping varicella caused by HSV, the differential diagnosis generally includes varicella-zoster virus infection, herpetic eczema and herpetic impetigo [24]. If necessary, causative agents in the blister fluid could be detected by nucleic acid testing [23]. Table 2 shows the main differential diagnoses of eczema coxsackium and some similar diseases.

## 6. Petechial/Purpuric Eruptions

**Figure 3:**
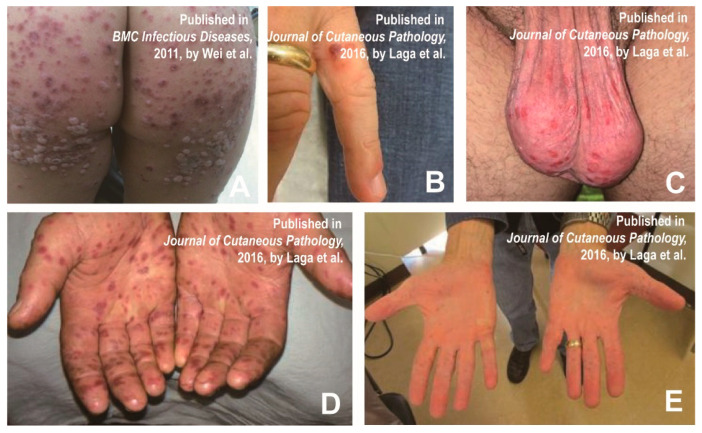
(**A**) A boy experienced prominent skin eruptions and vesicles on his buttocks. Reprinted/adapted with permission from Ref. [28]. 2011, Wei et al. (**B**–**E**) Erythematous purpura of adult patients and scattered blisters on the palms and scrotum. Reprinted/adapted with permission from Ref. [29]. 2016, Laga et al.

Petechial/purpuric eruptions consisting of relatively single blisters or concentrated blisters in some areas affected by atopic dermatitis or hemorrhagic or purpuric lesions are common atypical presentations of HFMD in children [12]. Some laboratory-confirmed CVA6-infected cases had a generalized purpuric rash around their mouth, trunk and/or neck, and feet, in addition to the typical rash on the hands. Profuse blistering and purpuric rash on a boy’s buttocks are shown in Figure 3A [28]. Laga et al. also reported atypical HFMD associated with CVA6 in adults, presenting erythematous purpura on the hands and scrotum (Figure 3B–E) [29]. In addition, leukocytoclastic vasculitis, as well as glove-and-stocking purpura (parvovirus infection), also have similar symptoms. HFMD is diagnosed clinically mainly by symptoms and pathogenic testing and does not require biopsy [29]. The histopathology of HFMD showed: spongiosis, neutrophilic exocytosis, massive keratinocyte necrosis, shadow cells in the upper epidermis, the vacuolization of basal cells, necrotic cells in follicles and sweat glands, dense superficial dermal infiltrates of CD3 lymphocytes, and strong granulysin expression [30]. Although pathological examination is not routinely used in clinical practice, leukocytoclastic vasculitis and glove and stocking purpura can also be distinguished by histopathology, as they are caused by other viral infections, subepidermal immunodeficiency or nutritional deficiencies [31,32]. Table 3 shows the main differential diagnoses of petechial/purpuric eruptions and some similar diseases.

## 7. Vesiculobullous Exanthema

**Figure 4:**
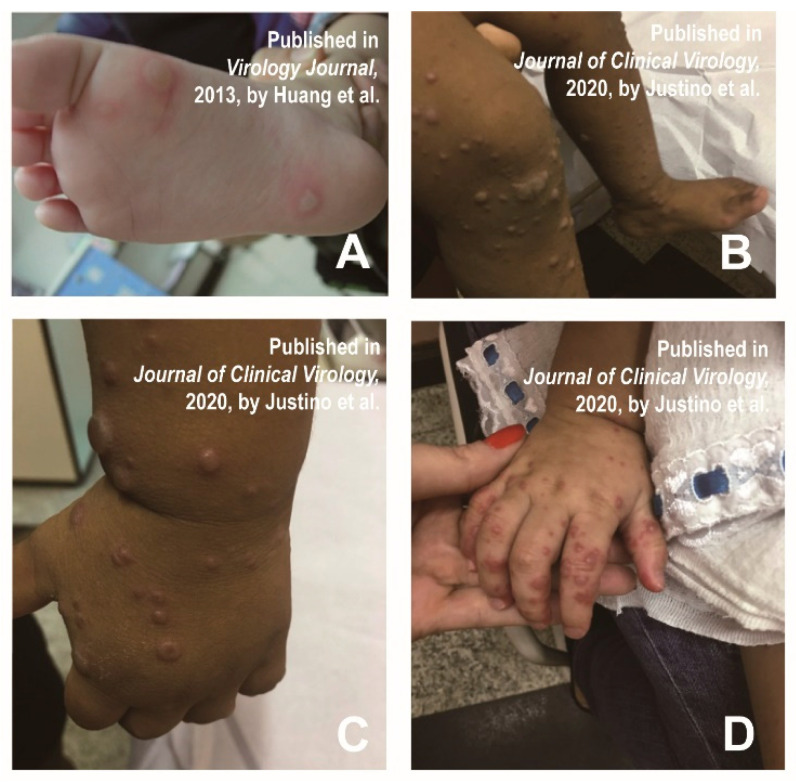
(**A**) Bullae were formed on a sole, accompanied by mild pruritus and prickling sensations. Reprinted/adapted with permission from Ref. [35]. 2013, Huang et al. (**B**–**D**) Bullous lesions on the extremities (knees and/or elbows) in patients with atypical HFMD. Reprinted/adapted with permission from Ref. [36]. 2020, Justino et al.

Since the spring of 2010, infantile HFMD infections from China and Taiwan have experienced an atypical skin injury, including large blisters on limbs, trunk or buttocks, and facial papules accompanied by fever, stomatitis and sore throat [35]. Successively, patients with bullous lesions were reported [12,35,36]. Huang et al. found that a 5-year-old male child had bullae on the bottom of his feet, causing slight itching and tingling (Figure 4A) [35]. Atypical lesions were most common on the face (41%), buttocks (31%), and trunk (29%). Bubble-like eruptions were also observed on the thigh, although it was rare in typical HFMD [35]. The large vesicle group was defined as vascular bullous lesions ≥ 1cm in diameter [35]. Mathes et al. found that 2 of the 80 patients presented vesiculobullous exanthema [12]. In a study of the clinical manifestation of 40 HFMD patients, 70% of them had bullous lesions on their faces, extremities (knees and/or elbows), buttocks, and necks lasting approximately two weeks (Figure 4B–D) [36]. 

In addition, the clinical presentation of vesiculobullous exanthema is similar to bullous impetigo, varicella and primary immune bullous, making it susceptible to misdiagnosis [16]. The clinicians can make a differential diagnosis by distinguishing the clinical manifestations, as well as symptom sites. Table 4 shows the main differential diagnoses of vesiculobullous exanthema and some similar diseases.

## 8. Tomato Flu 

Tomato flu, possibly caused by a variant HFMD-related pathogen, has been reported recently in India [43]. Recently, it was speculated that the etiological agent that caused ‘tomato flu’ was not a novel virus but an evolutionary CVA16 [43]. With the emergence of new pathogens or the recombination and evolution of classical viruses, the clinical characteristics of HFMD may also change a lot, which poses a challenge for correct and timely diagnosis. 

Unlike typical HFMD (mainly diagnosed according to physical examination and medical history), atypical cases may require laboratory examination [44]. Diagnosis can be confirmed by PCR (serum; oropharyngeal and skin swabs). If necessary, viral and bacterial cultures, direct antibody tests for viruses and skin biopsies can also be used to exclude other possibilities.

## 9. Geographical Distribution of Atypical HFMD

**Figure 5:**
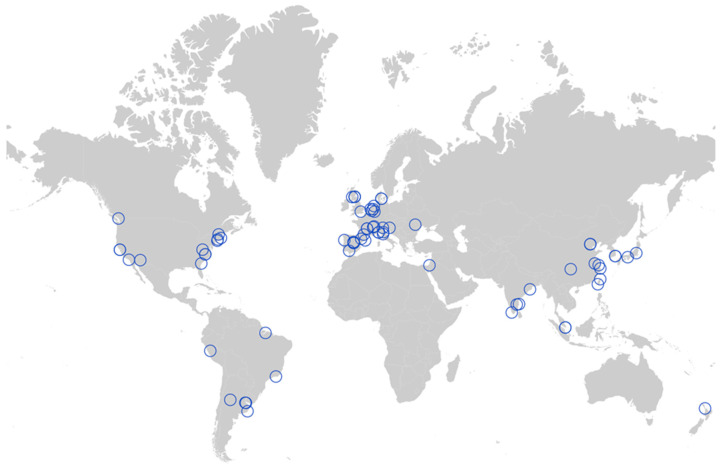
Geographical distribution of atypical HFMD. Data are visualized with the help of online website services (https://www.chiplot.online/map_plot.html, accessed on 3 January 2023).

Atypical HFMD has been reported worldwide, mostly in Asia, the Americas and Europe. (Figure 5) [45]. In China, most atypical HFMD cases were reported in coastal areas [46]. The subtropical and tropical monsoon climates characterized by high humidity and temperatures make them an environmental factor in HFMD epidemics [47]. Atypical HFMD also occurs in North America (the United States), South America (Brazil, Peru, and Argentina), Europe (Switzerland, the United Kingdom, the Netherlands, Denmark, Portugal, and Italy). Nevertheless, atypical HFMD is relatively rare in Africa, which may result from the fact that the viral agents causing atypical HFMD, such as CVA6, have not yet caused an outbreak in Africa. Of course, this is also related to the lack of real-time and effective public health surveillance in Africa.

## 10. Evolutionary Characteristics of Virus Strains Causing Atypical HFMD

**Figure 6:**
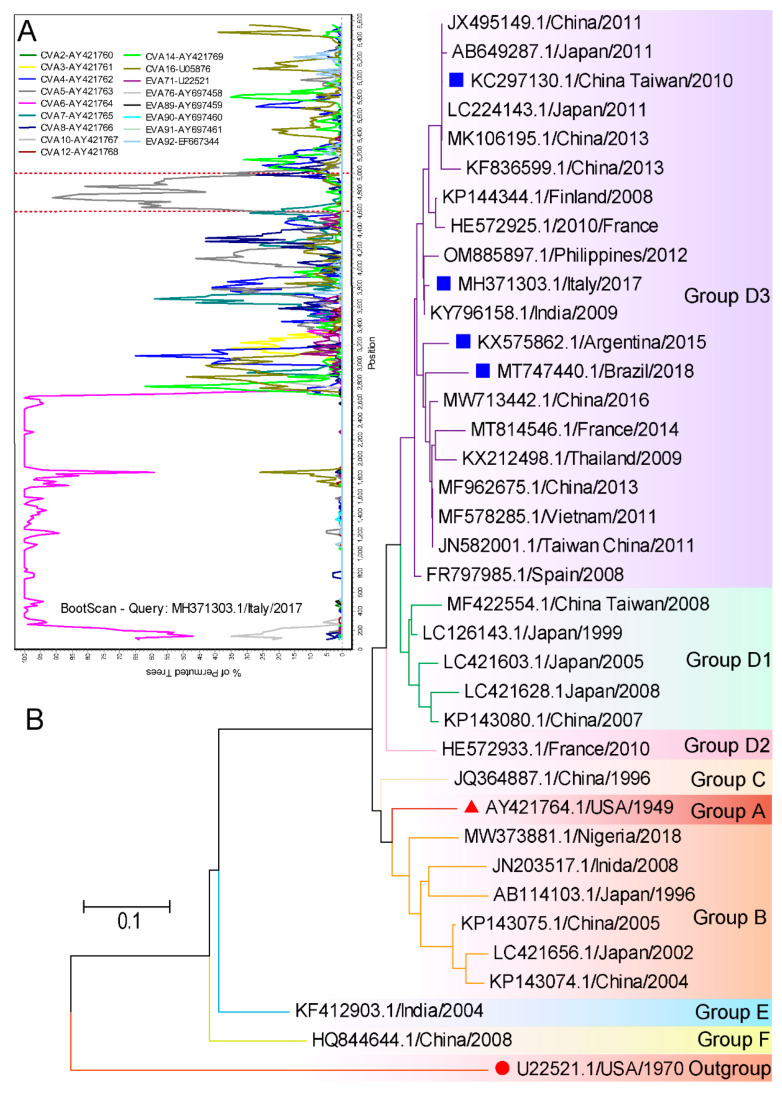
Evolutionary characteristics of CVA6 causing atypical HFMD available from GenBank were conducted applying MEGA 7 and Simplot 3.5.1 software. (**A**) The Bootscan analysis of MH371303.1/Italy/2017 based on the CDS region. The reference strains are represented in the legend. (**B**) Phylogenetic analyses of CVA6 based on a part of VP1 sequence (276 bp). The bootstrap test was performed with 1000 replications. All the strains are labelled using the following format: accession number/country of origin/year of isolation. The prototype strain of EVA71 marked with red circles indicates the outgroups. The prototype strain of CVA6 is marked as red triangles. The blue squares mark the representative strains causing atypical HFMD.

A variety of pathogens could cause atypical HFMD, but CVA6 has been commonly reported. Details such as atypical presentations caused by CVA6 are listed in Table 5. We applied Simplot and MEGA 7 for the evolutionary characterization of these strains from GenBank. The strains (MH371303.1) containing the entire gene coding sequence (CDS) were collected for recombination analysis. There was no recombination in the structural region, but possible recombination with CVA5 in non-structural regions marked by red dotted lines was detected (Figure 6A). CVA6 strains circulating worldwide can be divided into 6 genotypes designated as A to F, and the D genotype can be further subdivided into D1–D3 sub-genotypes. An analysis of these representative strains confirmed that all were sub-grouped into the genetic group D3. The D3 sub-genotype is the predominant genotype that circulates all over the world, particularly in Southeast Asia and Europe in recent years (Figure 6B). Thus, the virus strains causing atypical HFMD are very common. In addition to geographical location, conditions such as host immunity, socioeconomic status and ethnicity are suspected to contribute to the occurrence of atypical symptoms.

## 11. Summary and Prospective

Familiarity with the symptoms of the disease, including its atypical manifestations, is essential to make the correct diagnosis and treatment options. In view of the contagious nature of HFMD, a timely diagnosis can help avoid contact with the affected individual and decrease the risk of an outbreak. As an infectious disease primarily affecting children, there can be a delay in establishing the diagnosis in adult cases, which increases the risk of disease transmission. Therefore, we should give adequate attention to atypical HFMD in adults. Atypical HFMD is self-limiting with complete resolution usually within 7–14 days after disease onset. The treatment of atypical HFMD is usually symptomatic. Prompt recognition can reduce unnecessary treatment and hospital admission. The emergence of novel viruses or the recombination of common serotypes has brought considerable challenges to the timely differential diagnosis of HFMD. Clinicians must identify the wide range of cutaneous and mucosal alterations caused by the different EV types and prescribe appropriate diagnostic tests. Hence, establishing an adequate surveillance system for enteroviral diseases is a more appropriate public health intervention to combat HFMD. 

In this review, we summarized the current research on atypical manifestations of HFMD. Numerous studies have shown that the clinical presentations of HFMD caused by CVA6 infection are different from that of typical HFMD and may be more severe and widespread. The main clinical manifestations of atypical HFMD include Gianotti–Crosti-like eruptions, eczema coxsackium, petechial/purpuric eruptions and vesiculobullous exanthema. We also described the differential diagnosis of atypical HFMD in terms of rash characteristics, duration, common sites, and pathological features. However, some limitations of this study are worth noting. Firstly, we have only detailed the differential diagnosis of common diseases similar to atypical HFMD, as space is limited. Secondly, we likely underestimated the actual depth of the crisis caused by atypical HFMD due to the lack of real-time and effective public health surveillance in some regions, as well as the underdiagnosis of atypical HFMD. Despite these limitations, the present study provides a scientific guide for the timely diagnosis of HFMD and the prevention of disease progression.

## Figures and Tables

**Table 1:** Differential diagnosis of Gianotti–Crosti-like eruptions.

Differential Points	Gianotti–Crosti-Like Eruptions	Gianotti–Crosti Eruptions	Papulosquamous Urticaria	Erythema Infectiosum
Fever	More fever.	Low fever.	Generally no fever.	Fever.
Skin rash	Papules are symmetrically distributed on the face, arms and legs but relatively sparse on the trunk. Stomatitis of the oral mucosa [9,15].	Monomorphous, flat-topped, firm papules or papulovesicles. Located on the face, extensor surfaces of the extremities and buttocks. Symmetrical distribution. Lesions usually do not involve the palms, soles or mucous membranes of the hands [13].	Multiple bites and local pruritus. Grouped or disseminated urticarial papules, vesicles or wheals. Scratching can lead to ulcers and erosions.	The prodromal symptoms are low fever, malaise, sore throat, headache and nausea; after a few days, a red “slap on the cheek” facial rash appears. After two to four days, the facial rash subsides. Pink patches and blotches may appear in a lacy web-like pattern, most commonly on the extremities [16].
Itching	Mostly with pain and itching.	Mild to moderate itching.	Intense itching, possible epidermal peeling.	Paroxysmal itching, mostly worse at night.
Blisters	Mostly vesicles, blisters and large blisters.	Rarely seen.	Small blisters often present and can develop into large blisters.	Not yet been reported.
Recurrence	Presence of secondary features, peeling nails and peeling skin on hands and feet.	Rare recurrence.	Chronic or recurrent.	Disappears and reappears intermittently and disappears after a few days or weeks.
Duration	Usually one week.	Lasts significantly longer (2–8 weeks).	Can last from 2 to 12 days.	Around 1–6 weeks.
Pathogens	CVA6.	Epstein–Barr virus and hepatitis B virus Other viral factors such as CVA16, CVB4, CVB5, parvovirus B19, etc.	Often occurs after bites of insects such as mosquitoes, sandflies, bed bugs and fleas [17,18].	Caused by microvirus infection.
Prevalence age	Commonly seen in children and adolescents.	Commonly seen in children aged 1 to 6 years.	Commonly seen in children.	Usually involves children aged 3 to 12 years [19].

**Table 2:** Differential diagnosis of eczema coxsackium.

Differential Points	Eczema Coxsackium	Herpes Zoster	Herpetic Eczema
Fever	Usually accompanied by fever symptoms.	Fever is common.	Usually accompanied by fever.
Skin rash	Blisters and vesicles within the original dermatitis. The lesions are most severe in areas of eczema on bends, such as those on the arms and legs.	A characteristic narrow band-like rash on one side of the body from the spine to the front of the trunk. It may also occur on the face, eyes, mouth and ears. Mainly with skin inflammation and blisters [25].	Monomorphic eruptions of dome-shaped papulovesicles. After 1 or 2 weeks, blisters dry and form crusts that fill eroded pits. Vesicles may also be umbilicated, and the presence of ulcerations or slits is possible. Lesions are completely dried and healed after 2 to 6 weeks [26].
Pain or itching	Papules and blisters are mostly painful [27].	Severe and disabling pain.	Skin injuries are mostly painful.
Complications	Spontaneous regression and no serious systemic complications.	Postherpetic neuralgia, which is characterized by severe pain.	Including viremia, meningitis or encephalitis, hepatitis and septicemia due to Staphylococcus aureus infection.
Recurrence	In most patients, the rash shows signs of secondary bacterial infection.	Immunity develops after having shingles, and recurrences are uncommon in immunocompetent people.	Generally, no recurrence.
Duration	Lasts approximately 7–10 days.	Lasts approximately 2–4 weeks.	Lasts approximately 2–6 weeks.
Pathogens	Mostly CVA6.	Varicella zoster virus.	Herpes simplex virus.
Prevalence age	Commonly seen in children and adolescents.	Anyone infected with the primary varicella zoster virus can develop shingles.	Prevalent in children.

**Table 3:** Differential diagnosis of petechial/purpuric eruptions.

Differential Points	Petechial/Purpuric Eruptions	Leukocytoclastic Vasculitis	Glove and Stocking Purpura
Fever	Usually accompanied by fever symptoms.	May be accompanied by fever.	Fever.
Skin rash	Commonly found at the extremities.	Mainly involves the lower legs, but dependent areas such as the back may also be affected in hospitalized patients.	Edema, erythema and pruritic petechiae and papules in a distinct ‘glove and sock’ distribution. Usually associated with systemic symptoms, including fever, swollen lymph nodes, malaise, myalgia and arthralgia [33].
Itching	Not yet been reported.	May be completely asymptomatic. May present as burning pain.	Acral pruritus and pain [34].
Recurrence	Usually no recurrence.	Recurrence rate less than 20%.	Generally, no recurrence.
Duration	Mostly fades within 2 weeks.	Fades and slowly disappears within 2–3 weeks, leaving post-inflammatory hyperpigmentation.	Disappears within 1–2 weeks.
Histopathological features	The preferential involvement of the stratum granulosum and the upper half of the stratum spinosum. Intraepidermal vesiculation and reticular degeneration of cleared out spaces between cells. The keratinocytes showed marked eosinophilia with loss or diminution of the normal nuclear basophilic staining, resulting in a somewhat ‘ghost cell’-like appearance, consistent with necrosis. The lower third of the epidermis was also involved to a much lesser degree [29].	Evidence of neutrophilic infiltration within and around the vessel wall with signs of leukocytoclasia (disintegration of neutrophil nuclei into fragments or nuclear dust). Fibrinoid necrosis (fibrin deposition within and around the vessel walls). Signs of damage to the vessel wall and surrounding tissue (extravasated red blood cells, damaged endothelial cells) [31].	A mixed pattern of inflammation with interface and spongiotic changes. Parakeratotic scale with overlying basket-weave orthokeratosis. Within the epidermis, there were intraepidermal vesicles and Langerhans cell microabscess formations with scattered apoptotic keratinocytes. The underlying dermis showed a superficial perivascular lymphocytic infiltrate with mild edematous changes and extravasation of red blood cells [32].
Pathogens	CVA6.	Vasculitis of small vessels with inflammatory infiltration of neutrophils with leukocyte fragmentation.	Parvovirus B19.
Prevalence age	Commonly seen in patients over 5 years of age.	Tends to favor older patients and male patients.	Most prevalent in young adults.

**Table 4:** Differential diagnosis of vesiculobullous exanthema.

Differential Points	Vesiculobullous Exanthema	Bullous Impetigo	Varicella	Primary Immune Bullous (Take Bullous Pemphigoid, for Example)
Fever	Fever.	Generally, no fever.	Fever.	Not yet been reported.
Clinical Presentation	Blisters larger than 1cm in diameter. Widespread blistering and maculopapular eruptions. Beyond the typical palmar and plantar distribution of HFMD. Perioral, trunk and limb involvement also present.	Starts as a small blister and rapidly develops into a large blister with a loose surface. The blisters contain a clear or yellowish fluid which then becomes dark or pus-filled. The blisters rupture, resulting in a red ring of vesicles and scaly edges. Brown scab after the rupture of blisters [37].	The initial lesions often involve the scalp, face or trunk and appear as pruritic erythematous patches. Bright blisters appear on the skin, oval in shape, varying in size, surrounded by a red halo, itchy. Blisters, pustules and crusts may coexist in any one area of the skin [38].	Tense, serous or hemorrhagic bullae of 1–3 cm diameter can appear on erythematous or apparently normal skin. Urticarial or excoriated, eczematous plaques or prurigo-like lesions appear. Blisters evolve into eroded and crusted areas and then heal without scars. Symmetric distribution. Mucosal involvement is more common in the oral cavity [39].
Preferred site	Perioral, acral, and buttock predilection.	Lesions usually occur on the trunk, extremities and friction areas, such as the axillae, neck crease and nappy area. Often appear in well-defined clusters without any surrounding erythema or oedema.	A generalized rash with a concentration of skin blisters on the head, including the scalp and trunk, with fewer lesions on the extremities.	Include the lower abdomen, flexor surfaces of the limbs, groins and axillae.
Itching	Not yet been reported.	Sometimes itching and burning sensation, usually painless [40].	Usually accompanied by itching.	Almost all patients have itching.
Recurrence	Generally, no recurrence.	Generally, no recurrence.	No recurrence in general.	Not yet reported.
Duration	Lasting about two weeks.	Duration 2 to 3 weeks.	Duration 14 to 16 days.	May persist for some days to several months.
Pathogens	CVA6.	Staphylococcus aureus.	Varicella zoster virus.	The presence of circulating and tissue-bound IgG autoantibodies directed against BP180 and BP230 [41].
Prevalence age	Occurs mainly in children but also in adults.	Most often seen in infants and children, with 90% of cases occurring in children under 2 years of age [42].	Occurs mainly in children but also in adults.	Mostly occurs in the elderly population.

**Table 5:** Detailed information on virus strains associated with atypical HFMD.

Serotype	Atypical Presentation	Accession Number	Time	Country	References
CVA6	Fever with 48 h of evolution. Characteristic vesiculobullous and erosive eruptions, which primarily affect palms and soles. To a lesser extent, the rash affects the folds of the large joints, cheeks, perianal region and, in some cases, the whole body. Onychomadesis on the fingernails.	KX575862 - KX575865	2015	Argentina	[48]
CVA6	Bullae, which cause mild pruritus and prickling sensations. Atypical rashes of CVA6 spread on the forehead and temporal area of the face. Late presentation of atypical HFMD; severe desquamation presents on bilateral palms and soles. Onychomadesis occurs in the 2 weeks post-CVA6 infection.	KC297130 - KC297135	2010	Taiwan, China	[35]
CVA6	Adult involvement and delayed-onset palmar and plantar desquamation.	TW910141 - TW910142	2015	Taiwan, China	[49]
CVA6	Fever, cough, respiratory distress. Erythematous and pruritic lesions arise on the palms and soles of the feet, perioral region and ears, which further evolves to vesicles in hands, feet, mouth, and ears. Accompanied by a more extensive rash and skin damage. Blisters and scabs on hands, face and ears 6–14 days after the visit.	MT747440	2018	Brazil	[50]
CVA6	Fever. Erythematous vesicular lesions (mainly on hands and feet; mostly bilateral). Maculopapular purpuric lesions (on extensor surfaces and palm and soles or on the trunk). Lesions are painful, burning or itchy. Cutaneous lesions heal within 2–3 weeks with desquamation. Blisters and erosions of the hard palate are present in the mouth.	MH371303	2017	Italy	[44]

## Data Availability

All relevant data are within the manuscript and its Supporting Information files.

## References

[1] Solomon T., Lewthwaite P., Perera D., Cardosa M.J., McMinn P., Ooi M.H. (2010). Virology, epidemiology, pathogenesis, and control of enterovirus 71. Lancet Infect. Dis..

[2] Ooi M.H., Wong S.C., Lewthwaite P., Cardosa M.J., Solomon T. (2010). Clinical features, diagnosis, and management of enterovirus 71. Lancet Neurol..

[3] Merzel Sabovic E.K., Tockova O., Ursic T., Zgavec B., Dolenc-Voljc M. (2019). Atypical hand, foot, and mouth disease in an adult patient: A case report and literature review. Acta Derm. Alp. Pannonica Adriat..

[4] Bruning A.H., van der Sanden S.M., ten Hoedt A.E., Wolthers K.C., van Kaam A.H., Pajkrt D. (2015). An atypical course of coxsackievirus A6 associated hand, foot and mouth disease in extremely low birth weight preterm twins. J. Clin. Virol..

[5] Zhang Y.C., Jiang S.W., Gu W.Z., Hu A.R., Lu C.T., Liang X.Y., Hu Y.R., Zhu D.D., Xie L. (2012). Clinicopathologic features and molecular analysis of enterovirus 71 infection: Report of an autopsy case from the epidemic of hand, foot and mouth disease in China. Pathol Int..

[6] Lizasoain A., Piegas S., Victoria M., Da Silva E.E., Colina R. (2020). Hand-foot-and-mouth disease in uruguay: Coxsackievirus A6 identified as causative of an outbreak in a rural childcare center. J. Med. Virol..

[7] Mirand A., le Sage F.V., Pereira B., Cohen R., Levy C., Archimbaud C., Peigue-Lafeuille H., Bailly J.L., Henquell C. (2016). Ambulatory Pediatric Surveillance of Hand, Foot and Mouth Disease as Signal of an Outbreak of Coxsackievirus A6 Infections, France, 2014-2015. Emerg. Infect. Dis..

[8] Drago F., Ciccarese G., Gariazzo L., Cioni M., Parodi A. (2017). Acute localized exanthem due to Coxsackievirus A4. Infez. Med..

[9] Brandt O., Abeck D., Gianotti R., Burgdorf W. (2006). Gianotti-Crosti syndrome. J. Am. Acad. Derm..

[10] Kimmis B.D., Downing C., Tyring S. (2018). Hand-foot-and-mouth disease caused by coxsackievirus A6 on the rise. Cutis.

[11] Zhao T.S., Du J., Sun D.P., Zhu Q.R., Chen L.Y., Ye C., Wang S., Liu Y.Q., Cui F., Lu Q.B. (2020). A review and meta-analysis of the epidemiology and clinical presentation of coxsackievirus A6 causing hand-foot-mouth disease in China and global implications. Rev. Med. Virol..

[12] Mathes E.F., Oza V., Frieden I.J., Cordoro K.M., Yagi S., Howard R., Kristal L., Ginocchio C.C., Schaffer J., Maguiness S. (2013). “Eczema coxsackium” and unusual cutaneous findings in an enterovirus outbreak. Pediatrics.

[13] Leung A.K.C., Sergi C.M., Lam J.M., Leong K.F. (2019). Gianotti-Crosti syndrome (papular acrodermatitis of childhood) in the era of a viral recrudescence and vaccine opposition. World J. Pediatr..

[14] Marcassi A.P., Piazza C.A.D., Seize M., Cestari S. (2018). Atypical Gianotti-Crosti syndrome. Bras Derm..

[15] Losey N.A., Stevenson B.S., Busse H.J., Damste J.S.S., Rijpstra W.I.C., Rudd S., Lawson P.A. (2013). Thermoanaerobaculum aquaticum gen. nov., sp. nov., the first cultivated member of Acidobacteria subdivision 23, isolated from a hot spring. Int. J. Syst. Evol. Microbiol..

[16] Allmon A., Deane K., Martin K.L. (2015). Common Skin Rashes in Children. Am. Fam. Physician.

[17] Naimer S.A., Cohen A.D., Mumcuoglu K.Y., Vardy D.A. (2002). Household papular urticaria. Isr. Med. Assoc. J..

[18] Millikan L.E. (1993). Papular urticaria. Semin Derm..

[19] Vafaie J., Schwartz R.A. (2005). Erythema infectiosum. J. Cutan Med. Surg..

[20] Nahmias A.J., Froeschle J.E., Feorino P.M., McCord G. (1968). Generalized eruption in a child with eczema due to coxsackievirus A16. Arch. Derm..

[21] Ganguly S., Kuruvila S. (2016). Eczema Coxsackium. Indian J. Derm..

[22] Gin A., King E., Scardamaglia L., Orchard D. (2018). Eczema exacerbation caused by Coxsackie virus A6. Australas. J. Derm..

[23] Su H.J., Chen C.B. (2021). Eczema coxsackium. Med. J. Aust..

[24] Feder H.M., Bennett N., Modlin J.F. (2014). Atypical hand, foot, and mouth disease: A vesiculobullous eruption caused by Coxsackie virus A6. Lancet Infect Dis.

[25] Schmader K. (2018). Herpes Zoster. Ann. Intern. Med..

[26] Damour A., Garcia M., Seneschal J., Leveque N., Bodet C. (2020). Eczema Herpeticum: Clinical and Pathophysiological Aspects. Clin. Rev. Allergy Immunol..

[27] Ventarola D., Bordone L., Silverberg N. (2015). Update on hand-foot-and-mouth disease. Clin. Derm..

[28] Wei S.H., Huang Y.P., Liu M.C., Tsou T.P., Lin H.C., Lin T.L., Tsai C.Y., Chao Y.N., Chang L.Y., Hsu C.M. (2011). An outbreak of coxsackievirus A6 hand, foot, and mouth disease associated with onychomadesis in Taiwan, 2010. BMC Infect. Dis..

[29] Laga A.C., Shroba S.M., Hanna J. (2016). Atypical hand, foot and mouth disease in adults associated with coxsackievirus A6: A clinico-pathologic study. J. Cutan Pathol..

[30] Second J., Velter C., Cales S., Truchetet F., Lipsker D., Cribier B. (2017). Clinicopathologic analysis of atypical hand, foot, and mouth disease in adult patients. J. Am. Acad. Derm..

[31] Fraticelli P., Benfaremo D., Gabrielli A. (2021). Diagnosis and management of leukocytoclastic vasculitis. Intern. Emerg. Med..

[32] Zelman B., Muhlbauer A., Kim W., Speiser J. (2022). A rare case of papular-purpuric “gloves and socks” syndrome associated with influenza. J. Cutan Pathol..

[33] Halasz C.L., Cormier D., Den M. (1992). Petechial glove and sock syndrome caused by parvovirus B19. J. Am. Acad. Derm..

[34] Segura Saint-Gerons R., Ceballos Salobrena A., Gutierrez Torres P., Gonzalez Ruiz A., Gavilan Fernandez I., Martinez-Sahuquillo Marquez A. (2007). Papular purpuric gloves and socks syndrome. Presentation of a clinical case. Med. Oral. Patol. Oral. Cir. Bucal.

[35] Huang W.C., Huang L.M., Lu C.Y., Cheng A.L., Chang L.Y. (2013). Atypical hand-foot-mouth disease in children: A hospital-based prospective cohort study. Virol. J..

[36] Justino M.C.A., da S.M.D., Souza M.F., Farias F.P., Dos S.A.J.C., Ferreira J.L., Lopes D.P., Tavares F.N. (2020). Atypical hand-foot-mouth disease in Belem, Amazon region, northern Brazil, with detection of coxsackievirus A6. J. Clin. Virol..

[37] Hartman-Adams H., Banvard C., Juckett G. (2014). Impetigo: Diagnosis and treatment. Am. Fam. Physician.

[38] Arvin A.M. (1996). Varicella-zoster virus. Clin. Microbiol. Rev..

[39] Bagci I.S., Horvath O.N., Ruzicka T., Sardy M. (2017). Bullous pemphigoid. Autoimmun. Rev..

[40] Johnson M.K. (2020). Impetigo. Adv. Emerg. Nurs. J..

[41] Cole C., Borradori L., Amber K.T. (2022). Deciphering the Contribution of BP230 Autoantibodies in Bullous Pemphigoid. Antibodies.

[42] Brazel M., Desai A., Are A., Motaparthi K. (2021). Staphylococcal Scalded Skin Syndrome and Bullous Impetigo. Medicina.

[43] Chavda V.P., Patel K., Apostolopoulos V. (2023). Tomato flu outbreak in India. Lancet Respir. Med..

[44] Broccolo F., Drago F., Ciccarese G., Genoni A., Puggioni A., Rosa G.M., Parodi A., Manukyan H., Laassri M., Chumakov K. (2019). Severe atypical hand-foot-and-mouth disease in adults due to coxsackievirus A6: Clinical presentation and phylogenesis of CV-A6 strains. J. Clin. Virol..

[45] Aswathyraj S., Arunkumar G., Alidjinou E.K., Hober D. (2016). Hand, foot and mouth disease (HFMD): Emerging epidemiology and the need for a vaccine strategy. Med. Microbiol. Immunol..

[46] Zhang P., Zhang J. (2017). Surveillance on other infectious diarrheal diseases in China from 2014 to 2015. Zhonghua Liu Xing Bing Xue Za Zhi.

[47] Mao Y., Zhang N., Zhu B., Liu J., He R. (2019). A descriptive analysis of the Spatio-temporal distribution of intestinal infectious diseases in China. BMC Infect. Dis..

[48] Cisterna D.M., Lema C.L., Martinez L.M., Veron E., Contarino L.P., Acosta D., Freire M.C. (2019). Atypical hand, foot, and mouth disease caused by Coxsackievirus A6 in Argentina in 2015. Rev. Argent Microbiol..

[49] Chiu H.H., Liu M.T., Chung W.H., Ko Y.S., Lu C.F., Lan C.E., Lu C.W., Wei K.C. (2019). The Mechanism of Onychomadesis (Nail Shedding) and Beau’s Lines Following Hand-Foot-Mouth Disease. Viruses.

[50] De Sousa I.P., Giamberardino H.I., Raboni S.M., Debur M.C., de Lourdes Aguiar Oliveira M., Burlandy F.M., da Silva E.E. (2021). Simultaneous enterovirus EV-D68 and CVA6 infections causing acute respiratory distress syndrome and hand, foot and mouth disease. Virol. J..

